# Contamination of personal protective equipment during COVID-19 autopsies

**DOI:** 10.1007/s00428-021-03263-7

**Published:** 2022-01-06

**Authors:** Johanna M. Brandner, Peter Boor, Lukas Borcherding, Carolin Edler, Sven Gerber, Axel Heinemann, Julia Hilsenbeck, Atsuko Kasajima, Larissa Lohner, Bruno Märkl, Jessica Pablik, Ann Sophie Schröder, Julia Slotta-Huspenina, Linna Sommer, Jan-Peter Sperhake, Saskia von Stillfried, Sebastian Dintner

**Affiliations:** 1 Business Division of Safety, Security, and Compliance, University Medical Center Hamburg-Eppendorf, Hamburg, Germany; 2 Department of Dermatology and Venerology, University Medical Center Hamburg-Eppendorf, Hamburg, Germany; 3 DEFEAT PANDEMIcs Working Group, Hamburg, Germany; 4 Institute of Pathology, Rheinisch Westfaelische Technische Hochschule, Aachen University Hospital, Aachen, Germany; 5 General Pathology and Molecular Diagnostics, Medical Faculty, University of Augsburg, Stenglinstrasse 2, 86156 Augsburg, Germany; 6 Department of Legal Medicine, University Medical Center Hamburg-Eppendorf, Hamburg, Germany; 7 Institute of Pathology, University Hospital Carl Gustav Carus Dresden, Technical University of Dresden, Dresden, Germany; 8 Institute of Pathology, School of Medicine, Klinikum Rechts Der Isar, Technical University of Munich, Munich, Germany

**Keywords:** Autopsy, SARS-CoV-2, COVID-19, Personal protective equipment, Contamination

## Abstract

Confronted with an emerging infectious disease at the beginning of the COVID-19 pandemic, the medical community faced concerns regarding the safety of autopsies on those who died of the disease. This attitude has changed, and autopsies are now recognized as indispensable tools for understanding COVID-19, but the true risk of infection to autopsy staff is nevertheless still debated. To clarify the rate of SARS-CoV-2 contamination in personal protective equipment (PPE), swabs were taken at nine points in the PPE of one physician and one assistant after each of 11 full autopsies performed at four centers. Swabs were also obtained from three minimally invasive autopsies (MIAs) conducted at a fifth center. Lung/bronchus swabs of the deceased served as positive controls, and SARS-CoV-2 RNA was detected by real-time RT-PCR. In 9 of 11 full autopsies, PPE samples tested RNA positive through PCR, accounting for 41 of the 198 PPE samples taken (21%). The main contaminated items of the PPE were gloves (64% positive), aprons (50% positive), and the tops of shoes (36% positive) while the fronts of safety goggles, for example, were positive in only 4.5% of the samples, and all the face masks were negative. In MIAs, viral RNA was observed in one sample from a glove but not in other swabs. Infectious virus isolation in cell culture was performed on RNA-positive swabs from the full autopsies. Of all the RNA-positive PPE samples, 21% of the glove samples, taken in 3 of 11 full autopsies, tested positive for infectious virus. In conclusion, PPE was contaminated with viral RNA in 82% of autopsies. In 27% of autopsies, PPE was found to be contaminated even with infectious virus, representing a potential risk of infection to autopsy staff. Adequate PPE and hygiene measures, including appropriate waste deposition, are therefore essential to ensure a safe work environment.

**Supplementary Information:**

The online version contains supplementary material available at 10.1007/s00428-021-03263-7.

## Introduction

The results obtained from autopsies of those dying of severe acute respiratory syndrome coronavirus-2 (SARS-CoV-2) are of crucial importance to understanding coronavirus disease 2019 (COVID-19). Viral pneumonia with diffuse alveolar damage (DAD) is the most frequent cause of death in fatal cases of COVID-19. Beyond the dramatic changes in the lungs, the effect on multiple other organs is currently interpreted mainly as a systemic inflammatory reaction. Several authors have described endothelial impairment with consecutive activation of the coagulatory system [2, 5, 13, 20, 22, 26].

However, concerns about the safety of autopsy staff hampered the autopsy activities of surgical, forensic, and neuro-pathologists. Since the beginning of the pandemic, diverse authors and organizations have published many reports and guidelines on this topic [4, 11, 24].

A few studies, recently summarized by Meyerowitz et al. [14], have investigated the environmental viability of the virus in experimental conditions and real-world settings, including the domestic or clinical environments of SARS-CoV-2–positive persons. The presence of viable virus has been identified for up to 3 h in aerosols and 72 h on surfaces. Half-lives were calculated to be up to 6 h [19, 25].

In Germany, the Robert Koch Institute has recommended compliance with protection level 3, which requires wearing appropriate protective equipment when handling COVID-19 cadavers (surgical hood cap, eye/face protection with fully protective safety goggles or visors, filtering face piece [FFP] 2/3 masks, long-sleeved and impermeable protective clothing, waterproof apron, additional forearm protection, a second layer of latex/nitrile gloves with long cuffs, and appropriate shoes) [19].

Only a few reports have been published addressing topics related to the infectiousness of the cadavers of SARS-CoV-2–infected patients and the risk to autopsy staff. All those authors report detecting the virus through reverse transcription PCR (RT-PCR) in swabs taken from the airways at various time intervals after death [3, 7, 17, 18]. Schroeder et al. detected viral RNA on various body surfaces of the deceased as well as in body bags but found no viable viruses [21]. Pomara et al. report detecting viral RNA on the surfaces of autopsy tables, on autopsy room walls, and on face shields [18].

This study evaluated the extent of viral RNA contamination in the personal protective equipment (PPE) of autopsy staff during autopsies of COVID-19 patients, focusing particularly on the infectivity of samples found positive for SARS-CoV-2 RNA.

## Materials and methods

### Participating centers, case collection, and autopsy procedures

This study was conducted within the German framework of the DEFEAT PANDEMIcs initiative, which, among other objectives, aims to develop an operational and organizational basis for autopsy programs at the national level for pandemic preparedness. Four clinical pathology departments (Aachen [AA], Augsburg [AU], Dresden [DR], and Munich [MU]) and one department of legal medicine (Hamburg [HH]) participated in this study from January through May 2021. Four centers (AA, AU, DR, and HH) performed complete autopsies with the opening of all body cavities, including skulls. Minimally invasive autopsies (MIA) with ultrasound-conducted biopsies were performed at MU. (For technical details of the autopsies, see Supplementary Table 1.)

Written consent was obtained from the next of kin to perform the autopsies. The inclusion criterion for decedents was a confirmed diagnosis of SARS-CoV-2 infection as evidenced by a PCR test of the nasopharyngeal swab during the hospital stay and by either rapid PCR or antigen testing during the full autopsies. Three autopsies each from AU, DR, HH, and MU were included, and two cases were contributed by AA. The PPE for full autopsies comprised (from top to bottom) a head hood, goggles/face shield, FFP2/3 masks, coats, waterproof aprons, forearm protection, trousers, and rubber shoes/boots (see also Supplementary Table 1).

### Swabs: specimen collection and locations

**Fig. 1:**
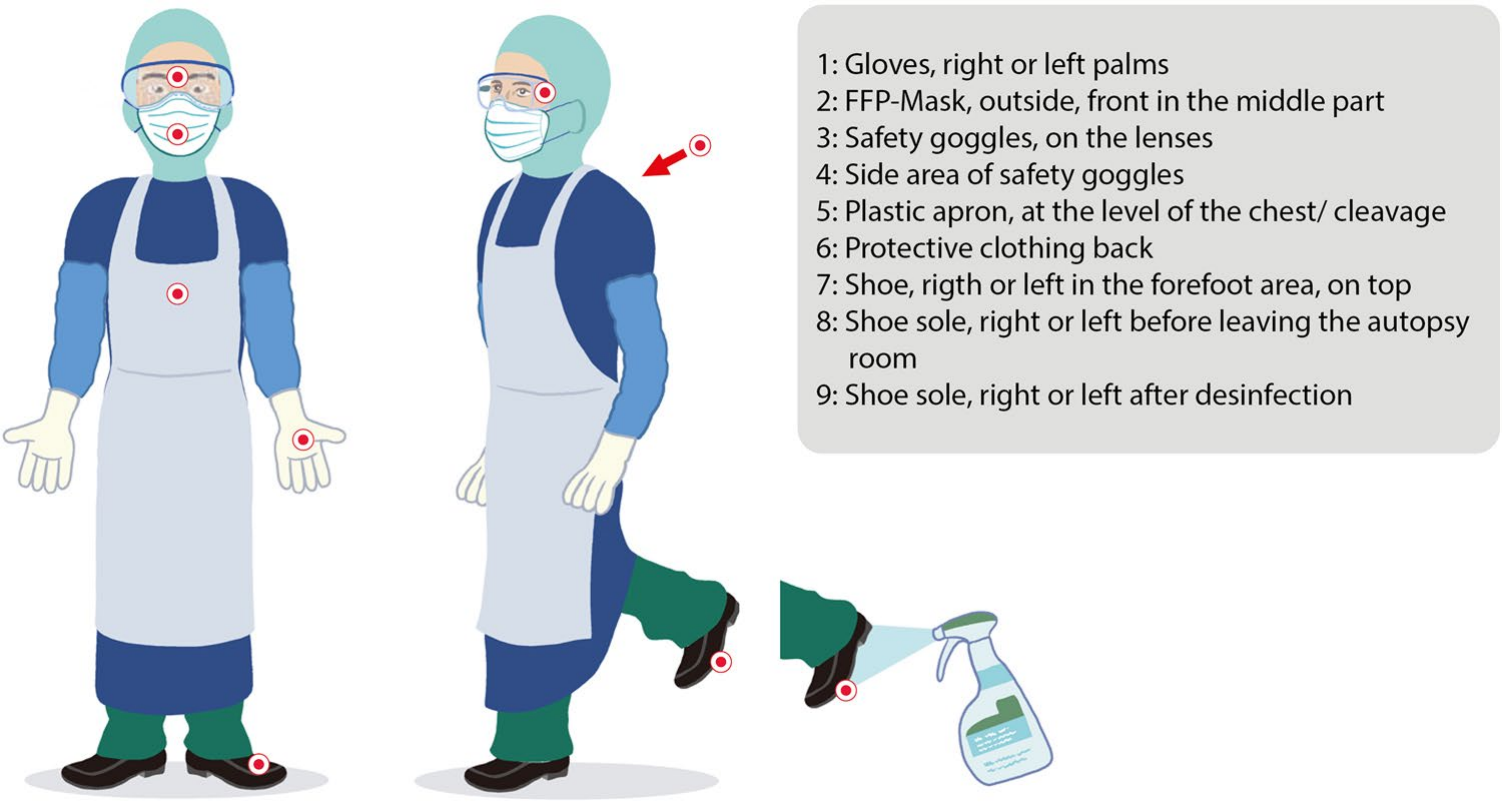
Schematic representation of the swab collection localizations

All the participating centers used an identical study protocol to collect swabs. Commercially available swab sets were used (COPAN eSwab B 80482CE, Mast Group, Reinfeld, Germany). Before swabbing, the tips were moistened with the transport medium, and then the PPE surfaces were thoroughly swabbed in a meandering manner for at least 15 s. The swabbed areas were not cleaned or disinfected before swabbing except in the case of autopsy no. 1, in which the gloves (but no other items) were cleaned with disinfectant wipes (Schuelke Safe & Easy Bagless) before swabbing. Finally, the tips were placed in the transport container. (Fig. 1 shows the swab locations.) Two swabs per location were taken next to each other, one for RT-PCR testing and one to test virus infectivity (virus isolation). In all the full autopsies, swabs were performed on the PPE of the autopsy-conducting physician and one autopsy assistant after the autopsy’s completion. (For an overview of the number of samples taken, see Supplementary Table 2.) The samples for RT-PCR testing were stored in refrigerators at 4 °C while swabs intended for eventual isolation of the infectious virus were frozen at − 80 °C. The PCR testing of all the samples was performed at AU. For samples from full autopsies that showed positive results in the PCR testing, the corresponding samples collected for viral isolation were sent to HH, where further PCR testing and virus isolation were performed.

In 8 of 11 autopsies (nos. 1, 2, and 6–11), visible contamination by blood and other bodily fluids was documented as follows: gloves, 16/16 positive (8 from physicians and 8 from assistants); FFP masks, 4/16; safety goggles, 3/16; aprons, 13/16). No other PPE items tested for viral contamination showed visible contamination.

In the MIAs, only the team member closest to the body of the deceased (the ultrasound-guiding physician) was evaluated by real-time RT-PCR testing.

To generate reference samples (positive controls), swabs from the plane-cut surface of the lungs were collected during each autopsy. In one case (AU2), the swab was taken from the bronchus and, in others, from lung biopsies (MIA, MU). (For an overview of the numbers of organ samples, see Supplementary Table 3.)

The time of storage before RT-PCR testing ranged from 0 to 19 days and that before viability testing from 34 to 89 days. There was no correlation between time of storage and outcome regarding RT-PCR or virus viability.

### Real-time RT-PCR

All the samples were processed and analyzed primarily at the Institute of Pathology and Molecular Diagnostics at the medical center in Augsburg. The method has been described in the literature [10], 13]. In brief, RNA was extracted using the Promega Maxwell 16 MDx system and the Promega Maxwell 16 LEV RNA Blood DNA Purification Kit (AS1290, Madison, WI, USA). Real-time PCR for SARS-CoV-2 was performed on the extracts with one-step multiplex RT-PCR for qualitative nucleic acid, targeting the SARS-CoV-2 ORF1ab, N protein, and S protein using the TaqPath COVID‑19 CE‑IVD RT‑PCR Kit (A48067, Thermo Fisher, Pleasanton, TX, USA). The RT-PCR was conducted using the QuantStudio 5 Dx Real-Time PCR instrument, and the data were analyzed and interpreted using QuantStudio™ design and analysis software (v.1.2x, Thermo Fisher, Carlsbad, CA, USA). Results with two or more positive targets were considered valid. A singular failure of the curve for the S protein constituted indirect evidence of the presence of a virus variant. Verification was carried out by comparing it with the results of the mutational diagnostics during the clinical stay.

### Cell culture and virus isolation

Duplicate swabs stored at − 80 °C to isolate the infectious virus from locations with RT-PCR positive swabs (as determined in AU) were transferred to the biosafety level 3 laboratory at the Institute of Medical Microbiology, Virology, and Hygiene at University Medical Center Hamburg-Eppendorf. To control the RNA integrity, confirmatory RT-PCR of the samples was performed as described [16]. Vero E6 cells were maintained and cultivated under standard conditions [6]. For virus isolation, 500 μl of swab medium was used, and infection was performed as described [6, 21]. Supernatants were harvested at 72-h post-infection, and the virus growth was analyzed as previously described [15]. The virus isolation experiments were restricted to samples from full autopsies.

### Statistics

The Mann–Whitney *U* test and one-way repeated measures ANOVA test were used to compare the data measured by order or rank. Spearman’s rank order correlation was employed to calculate correlations between the ranked data. Depending on the proportion numbers, tabulated nominal data were compared using either the chi-square test or Fisher’s exact test. A *p* value < 0.05 was considered significant. All the calculations were performed in the Sigmaplot 13.0 statistics package (Systat, San Jose, CA, USA).

## Results

### Case collection

Table 1 provides the case characteristics. Fourteen autopsies were included, of which three were conducted as MIAs. The median age of the deceased was 71 years (range: 52–91 years), with a male to female ratio of 1.8: 1. The postmortem interval (PMI) had a broad range (15–144 h; median 55 h). The median period from the first positive SARS-CoV-2 RT-PCR test to death was 10.5 days (range: 0–51 days). Autopsy duration ranged from 25 to 150 min.

**Table 1:** Demographic and autoptic data of all cases. *Rf*, reference organ (positive control); *Gl*, gloves; *n.a*., no data available; *n.i.*, no information concerning the viral lineage available; *evaluated during RT-PCR—loss of the S-curve as hint for variant of concern; **no testing prior to death; #tested positive in confirmatory RT-PCR at HH

	Autopsy/case no	Gender	Age [5-year intervals]	BMI [kg/m^2^]	Time between 1st positive test and death [days]	Time between death and autopsy [h]	Autopsy duration [min]	Location of death	Cause of death	Ct value reference sample at autopsy [N-gene]	Infectious virus	Variant of concern*
***Full autopsies***	1 (AA-1)	Male	70–74	22	28	38	25	Hospital	DAD—COVID-19	24	No	No
	2 (AA-2)	Female	85–89	23	25	64	25	Hospital	DAD—COVID-19	28	No	No
	3 (AU-1)	Male	85–89	25	8	15	150	Hospital	DAD—COVID-19	15	Rf /Gl	No
	4 (AU-2)	Female	75–79	36	13	40	150	Hospital	DAD—COVID-19	28	No	No
	5 (AU-3)	Female	90–94	16	19	36	150	Hospital	DAD—COVID-19	17	Rf	No
	6 (DR-1)	Male	60–64	50	7	96	120	Hospital	DAD—COVID-19	16	No	No
	7 (DR-2)	Male	80–84	36	10	120	120	Hospital	DAD—COVID-19	16	Rf/Gl	No
	8 (DR-3)	Male	65–69	35	3	72	120	Hospital	Combined hepatic and cardiac failure	14	Gl	Yes
	9 (HH-1)	Male	65–69	68	2	48	120	Outpatient	Sepsis due to pyelonephritis and pneumonia	15	No	No
	10 (HH-2)	Female	50–54	53	n.a	144	45	Hospital	DAD—COVID-19	32^#^	No	No
	11 (HH-3)	Female	70–74	30	0**	144	90	Outpatient	DAD—COVID-19	22	Rf	Yes
**MIA**	MU-1	Male	60–64	18	11	21	135	Hospital	n.a	20	/	Yes
	MU-2	Male	50–54	47	n.a	62	147	Hospital	Acute respiratory failure due to pneumothorax	Negative	/	n.i
	MU-3	Male	70–74	23	51	22	115	Hospital	n.a	Negative	/	n.i

### Real-time RT-PCR results of swabs from full autopsies

In total, 209 swabs for RT-PCR testing were performed for 11 full autopsies. Eleven of these samples were taken from the lungs/bronchi of the cadaver to serve as a reference (positive control) for each case (see also Supplementary Tables 2 and 3). All the lung/bronchus swabs (11/11) were positive.

**Fig. 2:**
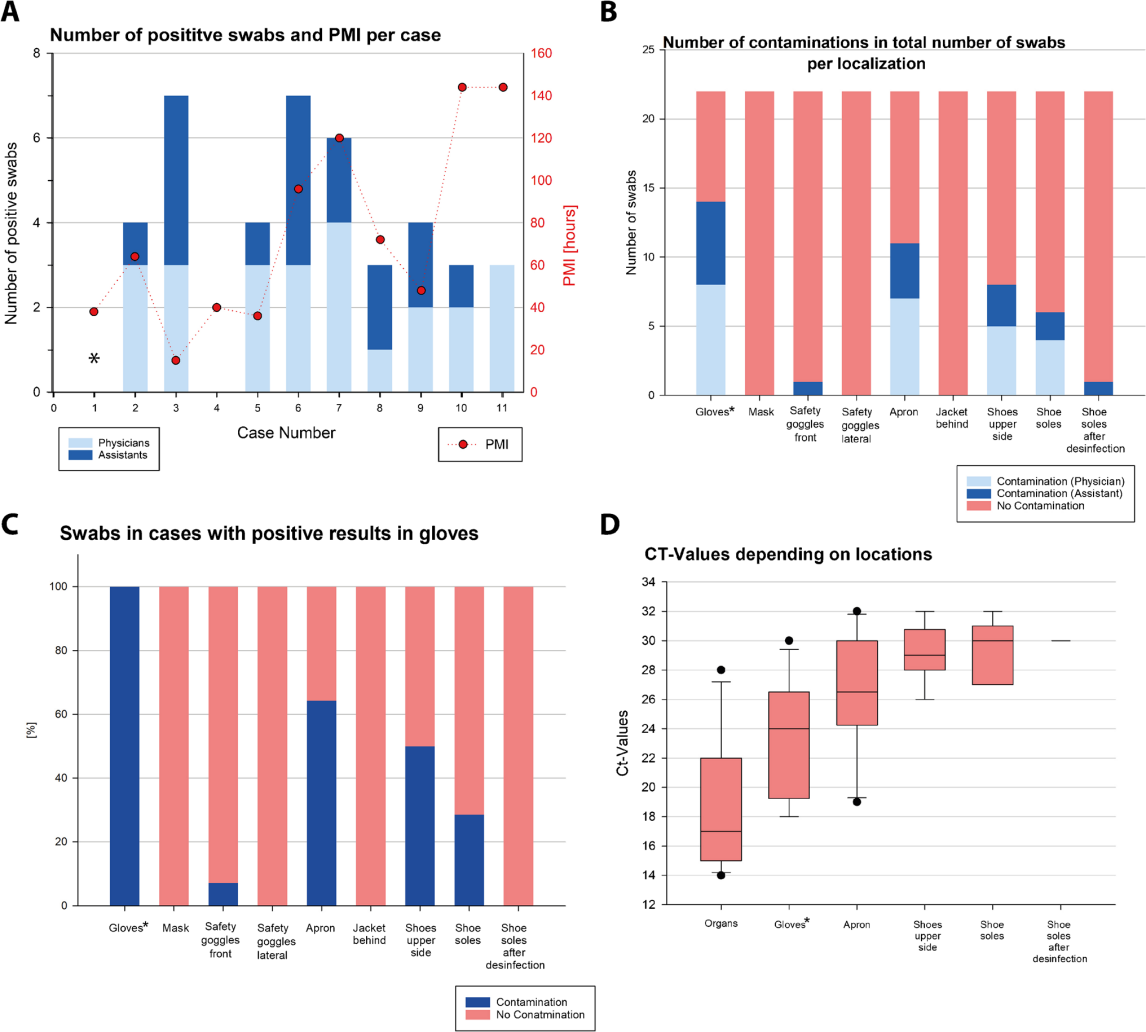
RT-PCR results from PPE. **a** Number of positive swabs per case divided according to physicians and assistants with corresponding PMI; **b** proportion of positive swabs from physicians and assistants in the various localizations; **c** results from PPE other than gloves in cases in which the gloves tested positive; **d** box plots of the Ct values by localization. Note: In one autopsy (AA1), swabs from the gloves were taken after disinfection

The remaining 198 swabs were collected from nine locations on the PPE of one physician and one assistant per autopsy (see also Supplementary Table 2). Of these, 41 (21%) were SARS-CoV-2 RNA positive, 24 from physicians, and 17 from assistants (Fig. 2A). In only two autopsies (2/11), all the PPE swabs were SARS-CoV-2 RNA negative (Fig. 2A) while RNA contamination was detected in 9 of 11 autopsies, with the number of SARS-CoV-2 RNA–positive PPE swabs per autopsy ranging from 3 to 7 (median: 4) (Fig. 2A). In one of the negative autopsies (AA1), the gloves were wiped with disinfectant at the end of the autopsy. No correlation was observed between the PMI and the number of RNA-positive PPE swabs (*p* = 0.503) (Fig. 2A). In addition, the total number of positive swabs per autopsy did not correlate with the cycle threshold (Ct) values of the lung/bronchus swabs (*R* =  − 0.51; *p* = 0.126) (Table 1).

The contamination at various locations is shown in Fig. 2B and C and Supplementary Fig. 1. Gloves were the most frequently contaminated item of PPE (14/22, i.e., 14 SARS-CoV-2–positive samples from 22 glove samples in total; 64%), followed by aprons (11/22; 50%), the tops of shoes (8/22; 36%), and shoe soles (6/22; 27%). Excluding the two glove samples disinfected before swabs were taken, 14 of 20 gloves were positive (70%). The front of the safety goggles was positive in 4.5% of the goggle samples while the FFP masks, sides of the safety goggles, and backs of the protective clothing were negative. In the 14 instances of positive gloves, the aprons were also positive in 9 cases (64%) and the tops of the shoes in 7 cases (50%) (Fig. 2C). A correlation trend was observed both between the contamination of gloves and that of aprons (*p* = 0.08) and between parallel RNA detection on the apron and on the tops of shoes (*R* = 0.38; *p* = 0.08). The tops of shoes also exhibited a correlation trend with a positive finding on the shoe soles (*R* = 0.39; *p* = 0.08) (Fig. 2B and C).

A highly significant difference (*p* < 0.001) was observed between the Ct values of the samples from the lungs or bronchi (median Ct: 17; range: 14–28) and those from the PPE (median Ct: 28; range: 18–32). In a sequel, the Ct values decreased to a highly significant extent in order from the lung/bronchus samples (positive controls) to the gloves, aprons, and shoes (Fig. 2D).

The evidence suggests a positive correlation between visible blood contamination and viral RNA contamination in gloves and aprons, as viral RNA contamination was observed only in association with visible blood contamination. However, this was not the case for goggles and shoes, in which viral RNA positivity was also found in samples without visible blood contamination (1/16 goggles, 4/16 shoes).

By contrast, some samples that were positive for visible blood contamination tested negative for viral RNA, including 4 of 16 gloves (including the 2 disinfected gloves), 6 of 16 aprons, 4 of 16 FFP masks, and 3 of 16 goggles.

### Real-time RT-PCR results of swabs from MIAs

In total, 30 swabs were performed for the three MIAs, including one lung control from each autopsy. The lung control tested positive for SARS-CoV-2 in one autopsy (MU1) and negative in the other two (MU2 and MU3) (Table 1). In MU1, viral RNA was detected on the gloves of the ultrasound physician while all the other swabs were negative. In MU2 and MU3, all the swabs tested negative for SARS-COV-2 RNA.

### Isolation of infectious virus from full autopsy samples

One hundred and ninety-eight swabs from PPE and 11 swabs from lungs/bronchi were taken in parallel with the swabs for RT-PCR and were stored at − 80 °C for assessment of infectivity by virus isolation. Virus isolation was performed on the 52 samples from locations at which the duplicate swab tested SARS-CoV-2 RNA positive at AU. Eleven of these samples were references obtained directly from the lungs/bronchi of the cadavers. The remaining 41 samples came from the positive PPE locations previously detected by RT-PCR.

**Fig. 3:**
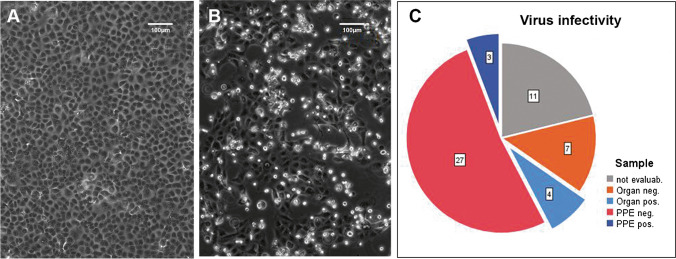
Exemplary representation of the cytopathic effect by SARS-CoV-2 in cell culture. **a** Uninfected Vero E6 cells grow to confluence in the cell culture; **b** infected Vero E6 cells already show a clear cytopathic effect at 48-h post-infection, characterized by rounding and detachment; **c** overview of swab samples from organs (lungs/bronchi) and from PPE that was positive or negative for successful virus isolation, reflecting virus infectivity

Virus isolation was successful in 4 of 11 (36%) lung/bronchus samples taken at three centers (AU, DR, and HH) (Fig. 3). Among the PPE samples, 11 of the 41 samples used for virus isolation tested negative in confirmatory RT-PCR performed at HH, suggesting the likelihood of RNA degradation, so they were excluded from the final calculation of the infectivity rate. In the interest of full disclosure, none of those samples yielded infectious virus.

Of the remaining 30 PPE samples, infectious virus was successfully isolated in three samples (3/30; 10%). All the positive samples came from gloves (1 assistant at AU, 1 assistant at DR, and 1 physician at DR). Thus, 21% (3/14) of the RNA-positive glove samples were infectious, and samples from 3 of 11 autopsies (27%) were infectious. In those autopsies, the median time between death and autopsy was 72 h (range: 15–120 h). The total number of positive swabs per autopsy did not correlate with the PMI (*R* = 0.049; *p* = 0.89).

On an autopsy-related basis, positive results for virus isolation from positive controls or PPE swabs were obtained in 4 of 6 autopsies with a lung/bronchus sample Ct value below 18, suggesting a high viral load, while only 1 of 5 cases with a lower virus concentration (Ct > 21) was infectious (for Ct values, see Table 1). However, this distribution trend did not reach significance (*p* = 0.242).

## Discussion

This study evaluated the extent and severity of SARS-CoV-2 contamination in the PPE of physicians and autopsy assistants who performed autopsies under real-world conditions on patients who died of COVID-19 at five German centers. The five centers represent four distinct geographic regions (north, south, east, and west), two medical disciplines (pathology and legal medicine), and two techniques (conventional complete autopsies and MIAs). Swabs were chosen as the method of generating samples for real-time RT-PCR and infectivity assessment in cell culture experiments as recently done by others [17, 18, 21].

Among the full autopsies, only those with a positive rapid SARS-CoV-2 diagnostic at the beginning of the autopsy were included. Consequently, all were SARS-CoV-2 RNA positive in the lung/bronchus samples used as the reference (positive control). Because rapid analysis was not available at MU, this criterion was not met, and the positive control sample (lung biopsy) tested positive in only one of the three MU autopsies. Thus, only one MIA autopsy could be evaluated. In that one positive case, the glove sample was positive, showing that PPE contamination could not be completely eliminated even in MIAs, but further investigations with a greater number of autopsies are necessary to determine its real extent.

Among full autopsies, SARS-CoV-2 RNA contamination of PPE items was found at a high frequency (9/11 autopsies). In only 2 of 11 full autopsies, neither the physician’s nor the assistant’s PPE was contaminated. Notably, in one of those two autopsies, the gloves were wiped with disinfectant at the end of the autopsy before the swabs were taken. This clearly affects the result of the glove samples in this autopsy, but an impact is unlikely at the other locations, as the disinfection took place at the very end of the autopsy. Pomara et al. report a considerably lower positivity rate of 5 of 16 autopsies (15.6% of 32 PPE samples taken) [18], but they evaluated only face shields. In our study, the front of safety goggles was contaminated in only a single case, resulting in an even lower positivity rate (1/22 goggle samples; 4.5%) than that of Pomara et al. while gloves (64%), aprons (50%), and shoes (tops: 36%; soles: 27%) were frequently positive. The distribution of the contaminated PPE items indicates that intensive mechanical contact is a cause of contamination. As expected, samples from the gloves tested positive most often, followed by swabs from the apron. Handling the cadavers and organs makes it difficult to avoid any contact, simply because the examiner’s distance from the specimen is necessarily minimal. The virus-contaminated material, very likely from gloves and aprons, reaches the shoes and floor, from which the shoe soles are also contaminated. This spatial sequence is supported by a stepwise increase in the Ct values, denoting a decreasing viral RNA load (Fig. 2D).

Due to the design of our study, we cannot draw conclusions regarding a possible additional contribution from aerosols, but all the samples from the FFP masks, the sides of safety goggles, and the backs of protective clothing tested negative for viral RNA, suggesting that aerosols are unlikely to be a relevant contamination source in the autopsy setting. However, we cannot exclude the possibility that the viral RNA may be less stable on those surfaces. Nonetheless, direct touch and splash represent the main threats of viral material transmission. This direct transmission is likely to be independent of the type of pathogen, substantiating the necessity of proper PPE and hygiene measures, including waste disposal during and after autopsies.

The results should be similar or even worse in other clinical settings, such as operating rooms, where staff experience close contact with the bodily fluids, secretions, and tissue of living Covid-19 patients as well as the virus-bearing aerosols produced by the patients’ breathing. A risk of contaminated PPE can also be expected in wards and intensive care units where, e.g., mucous secretions are aspirated.

In line with other reports investigating the persistence of viral material on and in people who have died of COVID-19 [3, 4, 8, 17, 18, 21, 23], a PMI of up to 144 h did not reduce the risk of PPE contamination with SARS-CoV-2 RNA. Our study did not aim to evaluate the stability of the virus on PPE over time, but Meyerowitz et al. have summarized the data on SARS-CoV-2 stability [14].

To assess infectivity, frozen swabs that had previously tested SARS-CoV-2 positive by RT-PCR were selected from all locations for further cultivation, including the reference lung/bronchus samples. Notably, only 41 of these 52 samples tested positive by confirmatory RT-PCR at HH; the samples that had tested negative by RT-PCR had to be excluded from the calculation of the overall infectivity rate. Retrospectively, it is difficult to identify the reason for this RT-PCR negativity, although RNA degradation during storage and transport may have been a factor. Remarkably, even though all the samples were shipped on dry ice by an experienced courier service, none of the negative samples came from HH, where the samples could be shipped in house. Notably, the non-evaluable samples were mainly from the shoes with high Ct values (7/11). In addition, it is conceivable that sampling the swabs at locations directly next to each other may have contributed to these differences, suggesting that the contamination may have been locally concentrated rather than diffuse.

Infectious virus was successfully isolated in 3 of 30 evaluable PPE samples (i.e., positive in confirmatory RT-PCR) but, notably, only from gloves. One might expect the viral load to be highest on gloves, which directly contact the organs, but the more fluid microenvironment on gloves at the time of sampling may also have contributed to the viral infectivity, in contrast to the dried-out viral material on other PPE items. To our knowledge, this is the first description of the isolation of infectious SARS-CoV-2 from PPE in an autopsy setting. Pomara et al. found SARS-CoV-2 RNA on 15.6% of face shields in an autopsy setting, but they did not investigate infectivity [18]. Regarding other extracorporeal surfaces, Schröder et al. found viral RNA on six body bags but detected no viable virus [21].

In the positive control lung/bronchus samples, the virus was successfully isolated in 4 of 11 cases (36%). There was a clear trend of a higher viability rate in cases with low Ct values (< 18) among the lung/bronchus samples, indicating that a higher viral load likely results in a higher probability of virus infectivity as has been previously shown [12]. There was no correlation between successful virus isolation and PMI. Also, Plenzig et al. report the isolation of viable viruses from lungs in two of four cases, independent of PMI [17].

Only the lung/bronchus samples and samples from gloves were infectious (with a higher infectivity rate in the lung/bronchus samples) while no infectious virus could be isolated from RNA-positive samples from aprons or shoes, which may indicate a degree of instability in SARS-CoV-2 as soon as it is transferred to inanimate surfaces. In an experimental setting, Haddow et al. investigated SARS-CoV-2 stability on diverse PPE materials (various face shields, coveralls, and 50/50 nylon/cotton ripstop fabric) and observed a PPE material–dependent reduction in plaque-forming units over 72 h [9]. Infectivity after transfer to surfaces may be higher or lower for other pathogens depending on the nature of the pathogen [1, 25].

In the context of the centers contributing to this study, our results led to a greater awareness in undressing and in the disinfection of PPE, especially gloves and shoes. In addition, it resulted in stronger observance of proper PPE waste disposal.

## Conclusion

This study found a considerable contamination rate of PPE during the autopsies of COVID-19 patients, and contamination occurred even in MIAs. Independent of the length of the PMI, SARS-CoV-2 RNA was detected in 21% of the samples taken from the PPE of 9 of 11 full autopsies. Gloves (64%), aprons (50%), and shoes (36%) showed the highest frequency of RNA contamination. Infectious virus was isolated from 21% of the RNA-positive glove samples (3/11 full autopsies).

In conclusion, the use of adequate PPE is mandated by several national and international bodies because the risk of infection during autopsy is a matter of reality, not a theoretical consideration. Together with hygiene measures, including appropriate waste disposal, PPE enables the safe performance of COVID-19 autopsies, which are essential for a better understanding of the disease. Pathologists investigating future infectious diseases are advised to select appropriate PPE and hygienic measures as a basis for conducting autopsies as an important source of new knowledge.

## Supplementary Information

Below is the link to the electronic supplementary material.

Supplementary file1. Supplementary Table 1. Technical and PPE details of the five centers contributing to the study. Supplementary Table 2. Overview of the number of PPE samples. Supplementary Table 3. Overview of the number of organ samples. (DOCX 18 KB)

Supplementary file2. Supplementary Figure 4. (a) Frequency of contamination of the PPE of physicians and assistants at various locations; (b) results from PPE other than gloves in cases in which the gloves tested positive. (JPG 1157 KB)

## Data Availability

The data may be requested from the corresponding author.
