# Supplementary material for: Contamination of personal protective equipment during COVID-19 autopsies
Source: Virchows Arch. 2022 Jan 6;480(3):519–28. doi: 10.1007/s00428-021-03263-7 (PMC8735722; doi:10.1007/s00428-021-03263-7)
Supplement: Supplementary file 1 — Supplementary file1. Supplementary Table 1. Technical and PPE details of the five centers contributing to the study. Supplementary Table 2. Overview of the number of PPE samples. Supplementary Table 3. Overview of the number of organ samples. (DOCX 18 KB) [file 428_2021_3263_MOESM1_ESM.docx]

**Suppl Table 1**

|  | **Aachen** | **Augsburg** | **Dresden** | **Hamburg** | **TU Munich** |
| --- | --- | --- | --- | --- | --- |
| Number of performed autopsies | 2 | 3 | 3 | 3 | 3 |
| Air exchange rate at least 10 times | X |  | X | X |  |
| Air treatment system with reverse flow | X | X |  | x | X |
| mask | FFP3X | FFP3 | FFP2 | FFP3 | FFP3 |
| Eye protection | X | X | X | x | X |
| Head hood | X | X | X | x | X |
| Coat | X | X | X | x | X |
| Gloves | X (double) | X (double) | X (double) | X (double) | X (double) |
| cut-resistant synthetic mesh/Kevlar gloves | X | X |  | no |  |
| waterproof apron | X | X | X | x | X |
| rubber shoes/boots | X | X | X | x | X |
| under PPE: surgical shirts, pants | X | X | X | x | X |
| Other | 2-step procedure, organ preparation after formalin fixation | Cartilage forceps for thorax,  IQ-Air Healthpro 250 NE room air purifier | Hand saw (non-electric) for skull trepination | Oscillating saw with suction | Hand drill for skull trephination |

TU = Technical Universitiy, FFP = filtering facepiece, PPE = personal protective equipment,

**Supplementary Table 2: Overview of PPE swabs**

| **Kind of autopsy** | **Number of autopsies** | **Number of persons tested per autopsy** | **Number of PPE localizations tested per person** | **Number of Swaps per localization** | **Total number of swabs from PPE** | **Total number of swabs from PPE investigated in real time RT- PCR** | **Total number of swabs stored for virus viability testing** | **Total number of swabs tested for virus viability** |
| --- | --- | --- | --- | --- | --- | --- | --- | --- |
| Full autopsy | 11 | 2  (1 physician, 1 technician) | 9 | 2 | 396 | 198 | 198 | 41 (Pre-Test PCR)  30 (final testing) |
| MIA | 3 | 1 (guiding physician) | 9 | 1 | 27 | 27 | - | - |

**Supplementary Table 3: Overview of organ swabs**

| **Kind of autopsy** | **Number of autopsies** | **Number of organ swabs per autopsy** | **Total number of organ swabs** | **Total number of organ swabs tested in real time RT- PCR** | **Total number of organ swabs tested for virus viability** |
| --- | --- | --- | --- | --- | --- |
| Full autopsy | 11 | 2 | 22 | 11 | 11 |
| MIA | 3 | 1 | 3 | 3 | - |
