# Supplementary figures and images for: Contamination of personal protective equipment during COVID-19 autopsies
Source: Virchows Arch. 2022 Jan 6;480(3):519–28. doi: 10.1007/s00428-021-03263-7 (PMC8735722; doi:10.1007/s00428-021-03263-7)

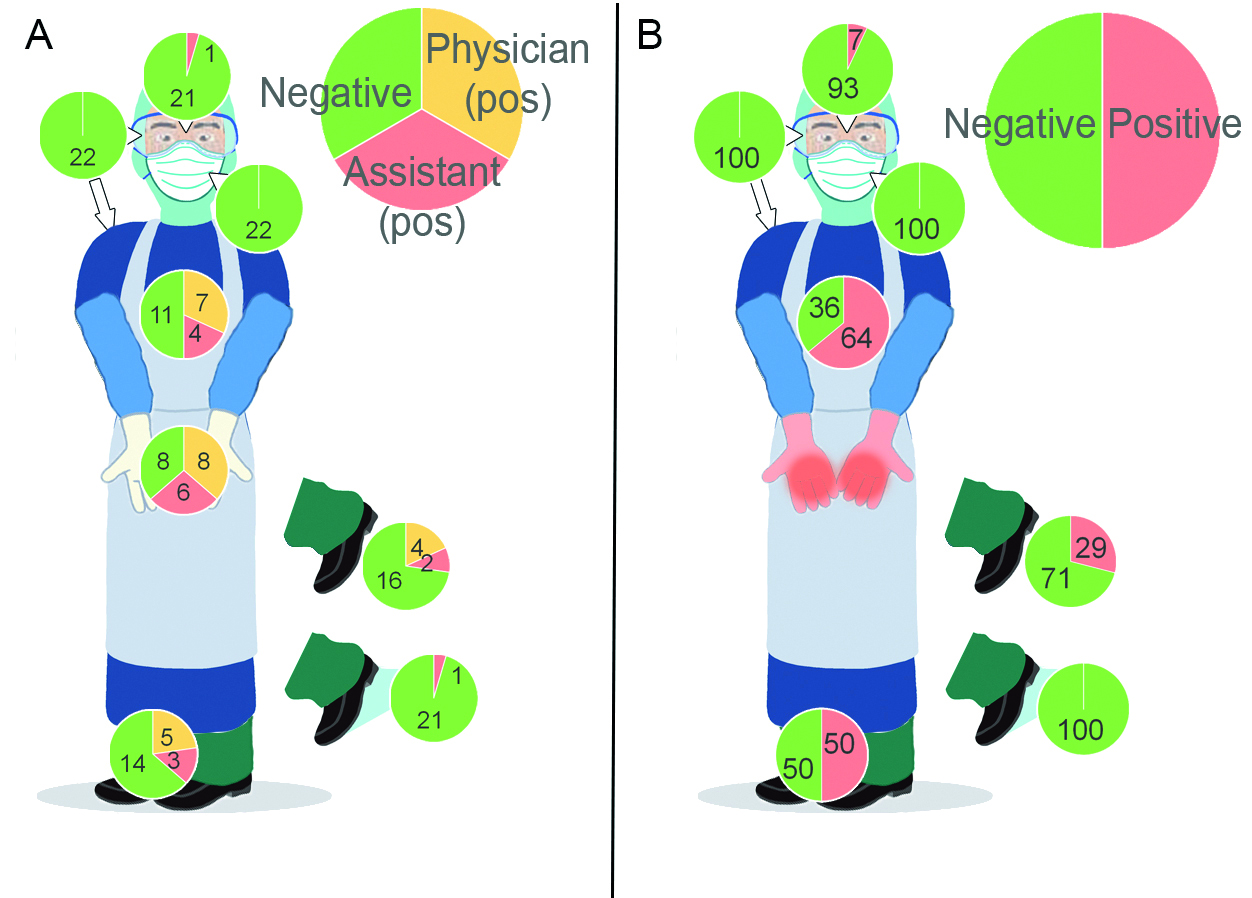

Supplement: Supplementary file 2 — Supplementary file2. Supplementary Figure 4. (a) Frequency of contamination of the PPE of physicians and assistants at various locations; (b) results from PPE other than gloves in cases in which the gloves tested positive. (JPG 1157 KB) [file 428_2021_3263_MOESM2_ESM.jpg]
